# Exploring Dielectric Constant and Dissipation Factor of LTCC Using Machine Learning

**DOI:** 10.3390/ma14195784

**Published:** 2021-10-03

**Authors:** Yu-chen Liu, Tzu-Yu Liu, Tien-Heng Huang, Kuo-Chuang Chiu, Shih-kang Lin

**Affiliations:** 1 Department of Materials Science and Engineering, National Cheng Kung University, Tainan 70101, Taiwan; 10809071@gs.ncku.edu.tw; 2 Hierarchical Green-Energy Materials (Hi-GEM) Research Center, National Cheng Kung University, Tainan 70101, Taiwan; 3 Material and Chemical Research Laboratories, Industrial Technology Research Institute, Hsinchu 31040, Taiwan; jill.t.y.liu@itri.org.tw (T.-Y.L.); TomHuang@itri.org.tw (T.-H.H.); ckc@itri.org.tw (K.-C.C.); 4 Core Facility Center, National Cheng Kung University, Tainan 70101, Taiwan; 5 Program on Smart and Sustainable Manufacturing, Academy of Innovative Semiconductor and Sustainable Manufacturing, National Cheng Kung University, Tainan 70101, Taiwan

**Keywords:** low-temperature co-fired ceramics (LTCCs), dielectric constant, dissipation factor, machine learning

## Abstract

Low-temperature co-fired ceramics (LTCCs) have been attracting attention due to rapid advances in wireless telecommunications. Low-dielectric-constant (*Dk*) and low-dissipation-factor (*Df*) LTCCs enable a low propagation delay and high signal quality. However, the wide ranges of glass, ceramic filler compositions, and processing features in fabricating LTCC make property modulating difficult via experimental trial-and-error approaches. In this study, we explored *Dk* and *Df* values of LTCCs using a machine learning method with a Gaussian kernel ridge regression model. A principal component analysis and k-means methods were initially performed to visually analyze data clustering and to reduce the dimension complexity. Model assessments, by using a five-fold cross-validation, residual analysis, and randomized test, suggest that the proposed *Dk* and *Df* models had some predictive ability, that the model selection was appropriate, and that the fittings were not just numerical due to a rather small data set. A cross-plot analysis and property contour plot were performed for the purpose of exploring potential LTCCs for real applications with *Dk* and *Df* values less than 10 and 2 × 10^−3^, respectively, at an operating frequency of 1 GHz. The proposed machine learning models can potentially be utilized to accelerate the design of technology-related LTCC systems.

## 1. Introduction

Low-temperature co-fired ceramics (LTCCs) have been attracting attention over recent decades due to rapid advances in wireless telecommunications, including the 5th generation (5G) tactile internet and the Internet of Things (IoT) [1]. LTCCs have characteristics that require sintering at temperatures of less than 1000 °C in order to be co-fired with electrode materials, such as Cu (melting point: 1083 °C), Ag (melting point: 961 °C), or Au (melting point: 1061 °C) [2]. LTCC devices provide a solution for integrating passive components, e.g., capacitors and resistors, with these electrodes into a three-dimensional module at the same time. A more recent review article from Sebastian et al. suggested that new LTCCs with ultra-low sintering temperatures (e.g., <700 °C) are becoming popular when pursing applications at a much lower temperature [3]. In recent years, the development of millimeter wave (mmWave) systems with typical frequencies above 24 GHz has led to performance benefits in 5G systems [4]. In the meantime, the increasing operating frequency from the current 4G systems at 3.5 GHz requires more reliable LTCC devices with a low dielectric constant (*Dk*), low dissipation factor (*Df*), and comparable mechanical strengths [1]. Wang et al. had pointed out that *Dk* and *Df* values should be lower than 10 and 2 × 10^−3^, respectively, for real applications at high frequency operation [5]. Ohsato et al. recently reviewed the current status and prospects of LTCC applications in microwave and mmWave telecommunications [6]. These low *Dk* and *Df* values enable a low propagation delay and high signal quality in 5G systems. 

LTCC fabrications are mostly based on glass-ceramic (GC) and glass/ceramic composite (GCC) in order to lower the sintering temperature [7]. Al_2_O_3_-based glass/ceramic composites are extensively used due to their good electrical and physical properties [8]. CaO-B_2_O_3_-SiO_2_-Al_2_O_3_/Al_2_O_3_ composites have been reported to be promising materials due to their low firing temperature and low dielectric loss [9]. B_2_O_3_-SiO_2_-Al_2_O_3_ glass and ZnO-B_2_O_3_-SiO_2_ glass/Al_2_O_3_ composites have been shown to exhibit a low dielectric loss and good mechanical and thermal performance [10]. La_2_O_3_-B_2_O_3_-CaO-P_2_O_5_ glass/cordierite [5] or La_2_O_3_-B_2_O_3_-CaO glass/LaBO_3_ composites [11] have recently been reported as potential candidates for real LTCC applications. Sebastian and Jantunen had made a thorough review on the material selections, fabrication methods, and properties of LTCCs [12]. The review suggests that LTCC fabrications involve modulating a wide range of glass, ceramic composition, and sintering conditions to meet the desired physical properties. The modulation is mainly carried out via experimental trial-and-error processes, and thus is time consuming and economically unfeasible. The wide ranges of input parameters make optimization even more difficult.

Theoretical modeling is an effective way to guide an experimental design. While there have been no existing models to properly simulate *Df* values, intrinsic *Dk* can be simulated via the density functional theory (DFT) in terms of polarizability. Peng et al. performed DFT calculation and the classical Clausius–Mossotti equation to model the dielectric constant of Li_2_(Mg_1-x_Ni_x_)SiO_4_ (x = 0.00–0.10) [13] and (Zn_1−x_Ni_x_)_3_B_2_O_6_ (x = 0.00–0.07) ceramics [14]. However, microstructure and processing features make extrinsic contributions which are not easily simulated. Therefore, a more reliable simulation method is required. Recently, machine learning methods are considered powerful tools to predict material properties which do not have existing physical models, e.g., effective charges in electromigration [15], permittivity of microwave dielectric ceramics [16], and dielectric constants of crystals [17]. Morgan and Jacobs had made a thorough review of recent applications of machine learning methods used in the field of materials science [18]. In this study, we thus employed the machine learning method to explore *Dk* and *Df* properties of LTCC. We used the glass phase content, ceramic filler content, and GC content as well as the processing features (e.g., calcination temperature and time) to fit the experimentally-determined *Dk* and *Df* data. Gaussian kernel ridge regression was used as it is powerful for interpolating data points which has fewer hyperparameters than typical deep learning method (e.g., neural network). Fitting to fewer hyperparameters is beneficial to a small-scale data set. We assessed the model using a cross-validation, randomized test, and cross-plot analysis. The results suggest that the proposed models had a reasonable predictive ability. We explored the composition and processing feature spaces to find potential LTCC systems with low *Dk* and *Df* values. The proposed models may serve as a quick guideline for new LTCC material design in future technology-related systems. To the best of the author’s knowledge, this is the first paper to use a machine learning method to explore *Dk* and *Df* of LTCC systems.

## 2. Methods

### 2.1. Data Set

The data set used in this study was provided by the Industrial Technology Research Institute (ITRI), Hsinchu, Taiwan. We refer to the database in this study as “ITRI-LTCC database.” The database is focused on exploring potential LTCC systems with low *Dk* and *Df* values that can be applied in real 5G applications using various GC, GCC, and fabrication methods. The database consists of glass phase content, ceramic filler content, GC content, processing parameters (e.g., calcination temperature and time), and *Dk* and *Df* measurements at various operating frequencies. The glass phases comprise two categories—(1) commercial glass and (2) and glass self-fabricated by ITRI. Commercial glasses consist of MgO-Al_2_O_3_-SiO_2_ glass (MASG), CaO-B_2_O_3_-SiO_2_ glass (CBSG), borosilicate glass (BG), and borosilicate glass + filler (BGF). For CBSG, the database includes two different compositions—one with higher SiO_2_ content and the other with higher B_2_O_3_ content. For the convenience of reference in the following discussion, we refer to the CBSG with higher SiO_2_ content as “CBSG-S,” and the one with higher B_2_O_3_ content as “CBSG-B.” Self-fabricated glass comprises mainly of MgO-Al_2_O_3_-SiO_2_-based glass (MAS), with a few data points for Li_2_O-Al_2_O_3_-SiO_2_-based glass and Al_2_O_3_-SiO_2_-based glass. MAS consists of different oxide compositions, i.e., different compositions of MgO, Al_2_O_3_, SiO_2_, and other relevant oxides. The ceramic filler and GC in the database are alumina and cordierite, respectively. A given self-fabricated glass might be chosen with a given mole percent and added into a glass or GCC comprising commercial glass, ceramic filler, and/or GC in order to tailor the microstructure and the corresponding properties. For example, 2 mol% MAS was added in a GCC composed of 30 wt.% alumina, 20 wt.% cordierite, and 50 wt.% CBSG-S. Processing parameters consist of three stages of calcination at various temperatures and times. We used *T1*, *T2*, and *T3* throughout this article to represent the first to third calcination temperatures, while used *time_1*, *time_2*, and *time_3* to represent the first to third calcination times, respectively (e.g., *T1* and *time_1* represent the first stage calcination temperature and time). *Dk* and *Df* values were measured at four different operating frequencies in the database, including 0.1, 1, 10, and 11 GHz, but mainly at 1 GHz. In the present study, the data measured at 1 GHz was pulled out to become an initial data set, which had a total number of 116 data points. Table 1 shows the feature information of these 116 data points. A principle component analysis (PCA) and k-means method from the scikit-learn library [19] were used for analyzing the data clustering in the initial data set. The proposed machining learning models for *Dk* and *Df* were thus developed on one of the clusters (see Section 3.1) with 63 data points. The proposed machine learning models are referred to as “the proposed models” in the following discussion.

Table 2 shows the feature information of the 63 data points after the PCA and k-means analysis used in developing the proposed models. The input features consisted of the glass phase content, GC (i.e., cordierite) content, ceramic filler (i.e., alumina) content, and the processing parameters. The glass phases were MASG, CBSG-S, CBSG-B, BG, and MAS. MAS had a constant composition of MgO, Al_2_O_3_, SiO_2_, and other relevant oxides. *T1* and *time_1* was constant at 750 °C and 3 h, respectively, while *T2, T3*, *time_2* and *time_3* remained variables. Note that, in Table 2, we defined two further features labeled as the *X* stage calcination reaction product (*TX_R*) and *X* stage calcination temperature and time product (*TX_time*), where *X* = second (2) or third (3). *TX_R* was defined as the calcination time multiplied by $\exp\left(\frac{-1}{\mathrm{calcination}\;\mathrm{temperature}}\right)$, while *TX_time* was defined as the calcination time multiplied by the calcination temperature. We found that these features showed a better model fit, for which the details are discussed in Section 3.1. 

### 2.2. Machine Learning Modeling

The machine learning model used in this work was the Gaussian kernel ridge regression (GKRR). The GKRR model uses the radial basis function kernel, where a hyperparameter *γ* represents the length scale between two given features. The Gaussian kernel has the form shown in Equation (1):

(1) $$k_{ij}=\exp\left(-\gamma {∥x_{i}-x_{j}∥}^{2}\right)$$

where $x_{i}$ and $x_{j}$ are given features vectors for LTCC *i* and *j*. $k_{ij}$ ranges from 0, which occurs when all the LTCCs *j* are infinitely far from LTCC *i* as measured by the kernel, to 1, which occurs when all the LTCCs *j* are infinitely close to LTCC *i* as measured by the kernel. The ridge regression uses a hyperparameter α as the coefficient of the L2-norm to penalize the fitting coefficients. The cost function *Φ* thus has the matrix form shown in Equation (2):

(2) $$\Phi ={\left|\left|Y-K\beta \right|\right|}_{2}^{2}+\alpha \beta^{T}K\beta$$

where Y is the target feature, K is the Gaussian kernel, and β are the kernel regression coefficients. The model analysis and exploration were primarily performed with the MAterials Simulation Toolkit for Machine Learning (MAST-ML, version 3.x, University of Wisconsin-Madison Computational Materials Group, Madison, WI, USA.) [20], an open-source Python package with scikit-learn [19] library to automate machine learning workflows and model assessments. The hyperparameters (*α*, *γ*) of the GKRR model were optimized using a genetic algorithm (GA) with the five-fold cross validation (CV) root-mean-square error (RMSE) as the scoring metric. Table 3 shows the optimized hyperparameters of *Dk* and *Df* models.

### 2.3. Model Assessment

A five-fold CV was used to assess the predictive ability of the model. The five-fold CV randomly partitioned the data set into five folds and took four of them as the training sub-dataset to build a GKRR model, with the remaining fold was used as the validation sub-data set. The CV process was repeated five times in one iteration, with each of the folds used exactly once as the validation dataset. The five-fold CV was repeated for 20 iterations. The 20 results were averaged to yield one prediction for each data point, and the average RMSE of these predictions was called the five-fold CV RMSE. The error bar for the five-fold CV RMSE was the standard deviation of the distribution of the RMSE values from the 20 iterations. The R^2^ score of the model was calculated, for which the method can be found elsewhere [15]. A randomized test was used to determine whether the model fitted to physical correlations that are not real. A randomized test involves randomly associating each *Dk* or *Df* with a given feature vector, but not the correct one. This gives a new data set that is exactly like the original one in terms of the actual values, where all the physical associations of the features and *Dk* or *Df* were removed. A five-fold CV was then performed for these randomized data to show the RMSE and R^2^ score. The interpolative quality of the model was examined using a cross-plot analysis, which shows how well the model predicted the target against a given variable with all the other variables held constant. A pair plot was generated using Python Library Seaborn [21].

## 3. Results and Discussion

### 3.1. Model Development

**Figure 1:**
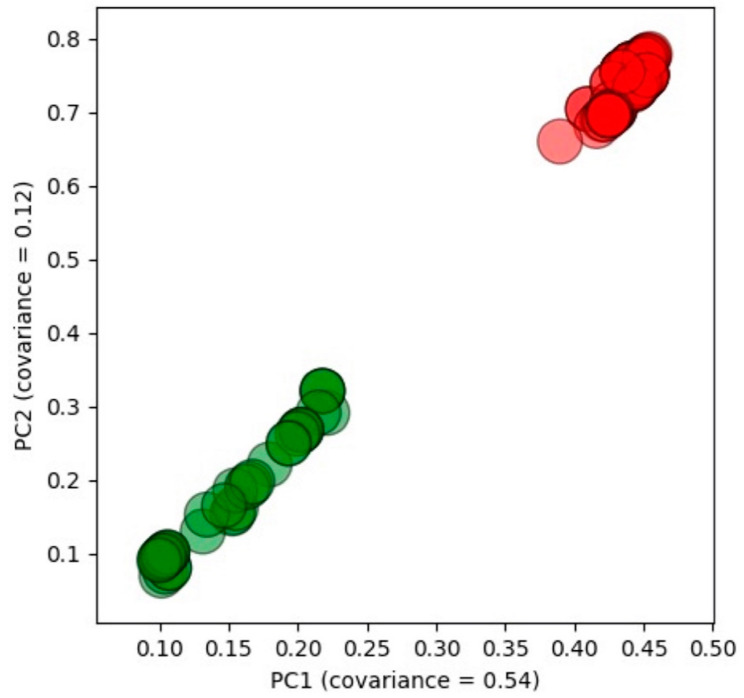
Cluster plot of the initial data set.

**Figure 2:**
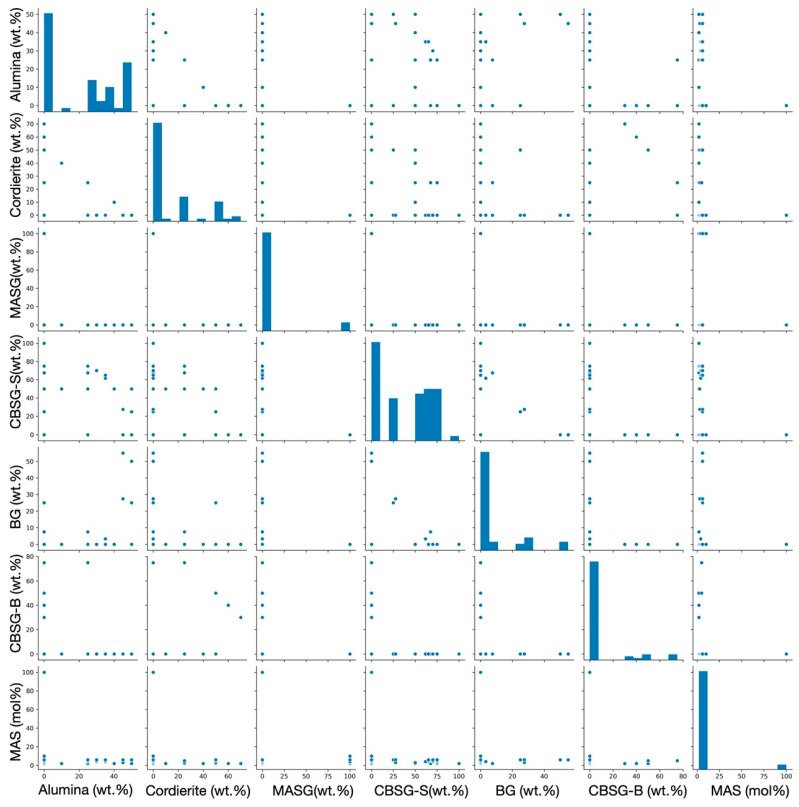
Pair plot of the input glass phase, ceramic filler, and GC content distribution.

We intended to use the initial data set with 116 data points (see Table 1) to develop machine learning models for *Dk* and *Df*; however, we failed to develop decent models. In developing the model for *Dk*, the best five-fold CV RMSE was 0.64; the CV RMSE over the standard deviation of the data set (RMSE/σ) was 0.12, and the R^2^ was only 0.28. The optimal input feature vector was found to be *T1*, *T2*, *T3*, alumina, cordierite, MASG, CBSG-S, CBSG-B, BG, BGF, and MAS. Two issues were raised: (1) The model did not capture the calcination time; (2) in the initial data set, because MgO, Al_2_O_3_, SiO_2_, and the other relevant oxides content were not constant in MAS, the oxide composition should also be considered as part of the input features. Nevertheless, the model did not capture the oxide composition either. Similar circumstances were found when developing the *Df* model. We suspected that there was data bias due to clustering originated from the unbalancing weight of the limited data sampling. To examine the clustering issue, we performed the PCA and k-means method and show the cluster plot in Figure 1. There were two groups that were apparent in the cluster plot, i.e., the green group and the red group. Red points were found much closer to the clustering center than the green one. After careful examination of the data set, we found that the red group shared the same oxide composition in MAS, while the green group did not. In other words, the oxide composition in MAS could be held constant when using the red group data as the training data set. On the other hand, the first stage of the calcination temperature and time (i.e., *T1* = 750 °C, *time_1* = 3 h), as well as the BGF content (i.e., BGF = 0 wt.%) were also constant in the red group. Figure 2 shows a pair plot of the glass, ceramic filler, and GC content distribution in the red group data. The scattered points in the plot show how the data sampled along the composition spaces. We thus pulled out the red group data with a total number of 63 data points to build *Dk* and *Df* models (see Table 2 for the feature information). By using a cluster plot analysis, feature dimensions and complexity were reduced although we paid the price of losing some data. Nevertheless, it was still better than having models that were not consistent with the actual experimental fabrication process. 

**Figure 3:**
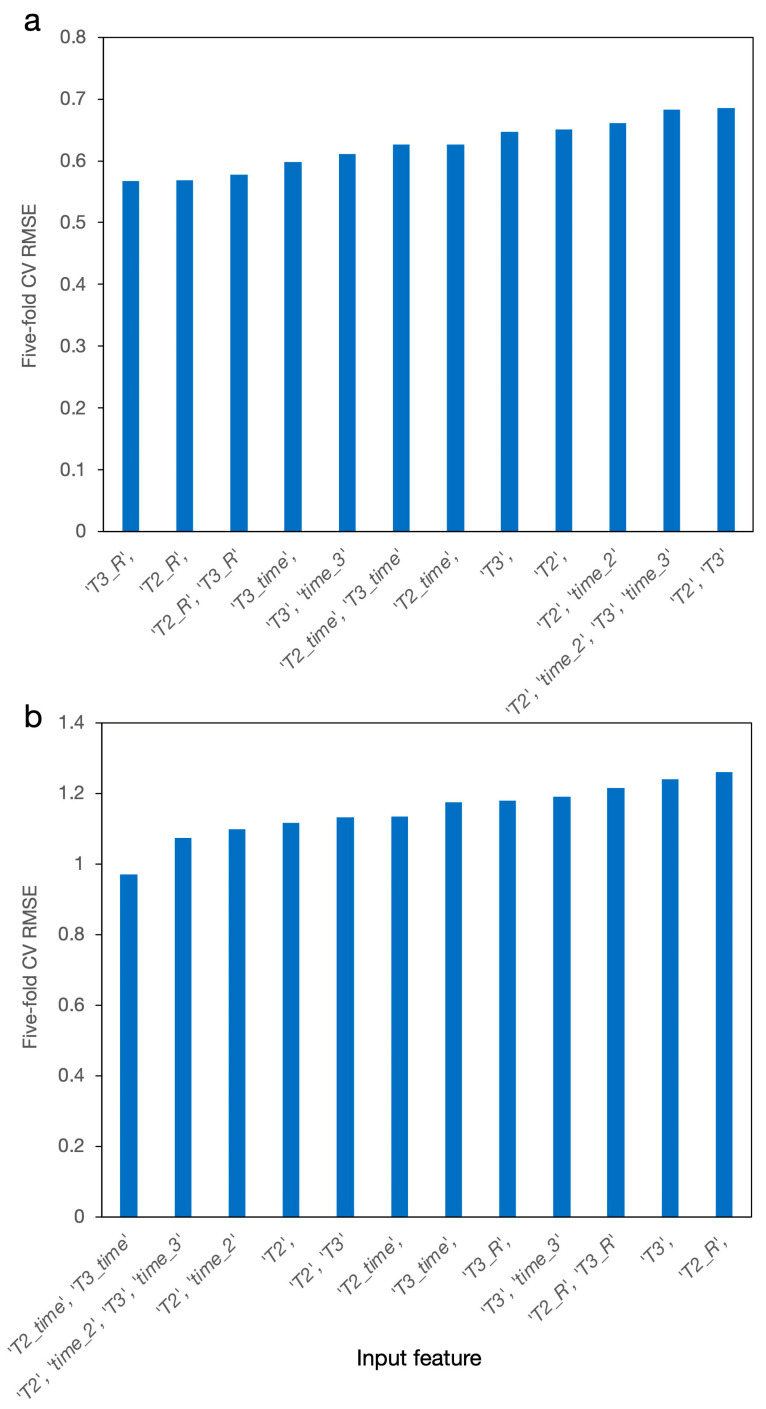
Five-fold CV RMSE vs. input feature to the model built for (**a**) *Dk* and (**b**) *Df*. The x-axis was sorted based on the CV RMSE values.

When developing *Dk* and *Df* models using the red group data, we firstly explored the optimal input feature vector. Figure 3 shows the five-fold CV RMSE vs. the top 12 input features, where the plot was sorted based on the CV RMSE values. The x-axis shows the input feature used in building the model. We showed only the processing parameters in the x-axis label because the glass, ceramic filler, and GC content (i.e., alumina, cordierite, MASG, CBSG-S, CBSG-B, BG, and MAS) remained the same in each feature vector. For instance, “*T3_R*” in Figure 3a means we used *T3_R*, alumina, cordierite, MASG, CBSG-S, CBSG-B, BG, and MAS as the input for building *Dk* model. This input feature showed the lowest CV RMSE for *Dk* model. “*T2_time*, *T3_time*” in Figure 3b means we used *T2_time*, *T3*_time, alumina, cordierite, MASG, CBSG-S, CBSG-B, BG, and MAS as the input for building *Df* model and that this input feature showed the lowest CV RMSE for *Df* model. Note that, in Figure 3a, even though “*T2_R*, *T3_R*” showed the third-lowest CV RMSE, the difference in the RMSE between the lowest one was only 0.01. On the other hand, using the “*T2_R*, *T3_R*” feature set would be more consistent with actual experimental conditions. We felt that the model was actually capturing the real processing parameters, but due to the limited sampling of the data set, a numerical fitting issue occurred. Therefore, we manually selected the *T2_R*, *T3_R*, alumina, cordierite, MASG, CBSG-S, CBSG-B, BG, and MAS as the input for building *Dk* model. We did our best to explore as many processing parameter combinations as possible to see if the models could be further improved. For instance, we attempted to multiply the *T2* term by the *T3* term to see if coupling between the processing temperatures occurred. Nevertheless, it turned out that these CV RMSEs were not further improved. Overall, the designed features of *T2_R*, *T3_R*, *T2_time*, and *T3*_time worked better than using *T2*, *T3*, *time_2*, and *time_3* directly. This may suggest some underlying physics in terms of *Dk* and *Df* function that works against the processing parameters although the physics is not easily examined directly using a machine learning model. Using “*T2_R*, *T3_R*” or “*T2_time*, *T3_time*” also helped to reduce the number of feature dimensions and helped avoid the overfitting issue. In the meantime, the models built on these features were consistent with the real processing circumstances.

### 3.2. Model Assessment

**Figure 4:**
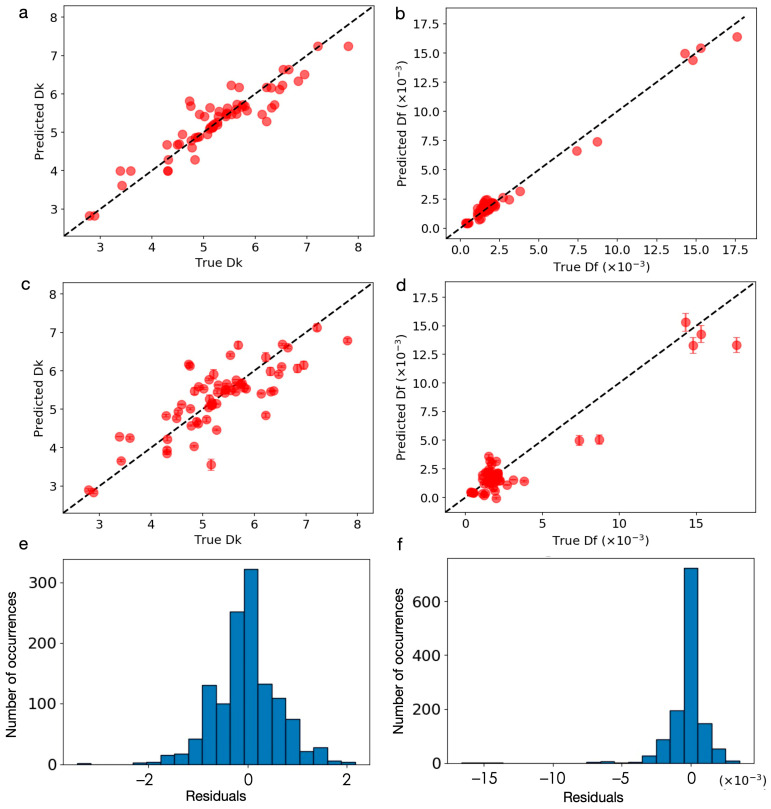
Parity plot of the (**a**,**b**) full-fit and (**c**,**d**) five-fold CV for (**a**,**c**) *Dk* and (**b**,**d**) *Df* models. (**a**) RMSE = 0.37, RMSE/σ = 0.38, R^2^ = 0.82. (**b**) RMSE=0.39 × 10^−3^, RMSE/σ = 0.11, R^2^ = 0.99. (**c**) CV RMSE = 0.59, CV RMSE/σ = 0.61, R^2^ = 0.57. (**d**) CV RMSE=1.12 × 10^−3^, CV RMSE/σ = 0.31, R^2^ = 0.91. The residual plot of the five-fold CV for (**e**) *Dk* and (**f**) *Df* model.

Figure 4a,b show the parity plots of the full-fit for the proposed *Dk* and *Df* models fitted to the optimal features, respectively. The RMSE was 0.37, the RMSE/σ was 0.38, and the R^2^ was 0.82 for *Dk* model. The RMSE was 0.39 × 10^−3^, the RMSE/σ was 0.11, and the R^2^ was 0.99 for *Df* model. Figure 4c,d show the parity plots of the five-fold CV for the proposed *Dk* and *Df* models, respectively. The CV RMSE was 0.59, the CV RMSE /σ was 0.61, and the R^2^ was 0.57 for the *Dk* model. The CV RMSE was 1.12 × 10^−3^, the CV RMSE/σ was 0.31, and the R^2^ was 0.91 for the *Df* model. Overall, the RMSE/σ values were all less than one, which suggests that our models captured the complex *Dk* and *Df* properties by providing information only for the glass, ceramic filler, GC content, and the processing parameters. Figure 4e,f show the residual plots of the five-fold CV, for which the results showed an approximately normal distribution. This suggests that the choice of the GKRR model was appropriate.

**Figure 5:**
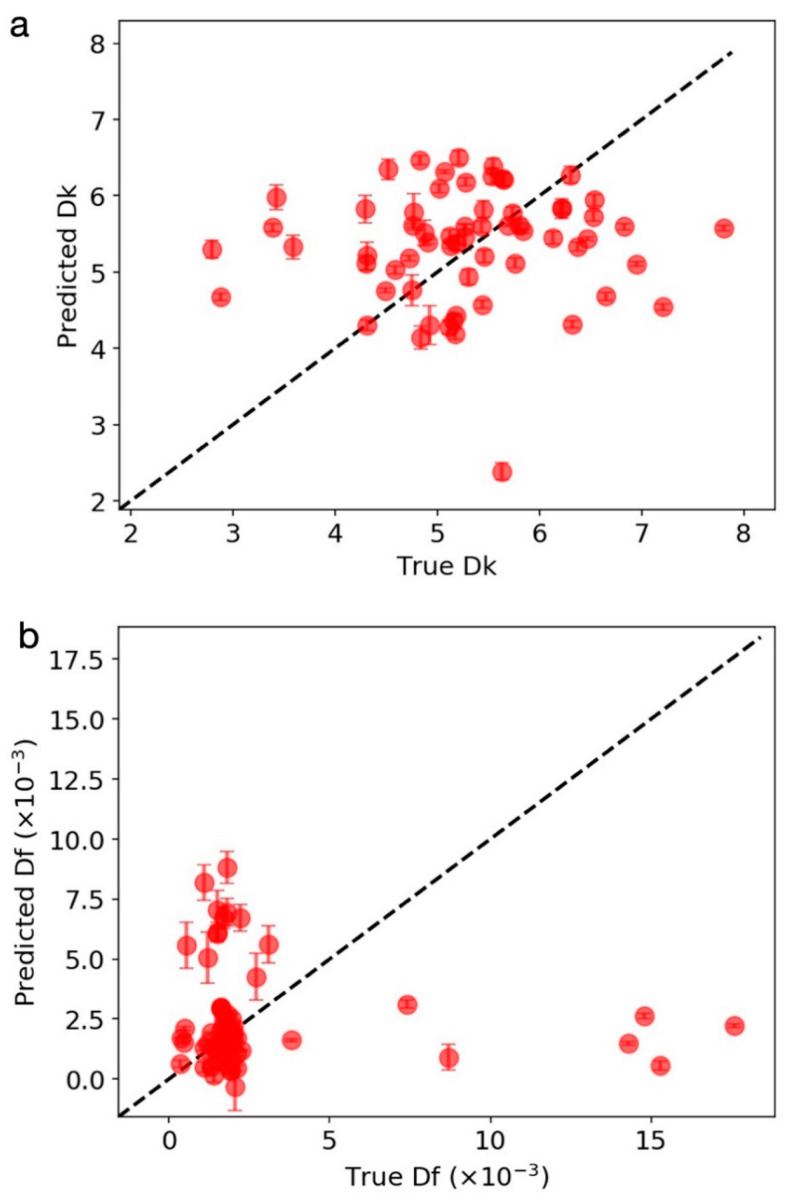
Parity plot of the randomized test for (**a**) *Dk* and (**b**) *Df* models. (**a**) RMSE = 1.19, RMSE/σ = 1.22, R^2^ = −1.58. (**b**) RMSE=4.33 × 10^−3^, RMSE/σ = 1.21, R^2^ = −0.94.

Due to a rather small scale of the data set with limited sampling (see Figure 2), it is conceivable that the model may not fit the correct physical correlations. To investigate this, we performed a randomized test [15]. Figure 5a,b shows the parity plots of the randomized tests for *Dk* and *Df* models, respectively. The RMSE was 1.19, the RMSE/σ was 1.22, and the R^2^ was −1.58 for *Dk* model in the randomized test. The RMSE was 4.33 × 10^−3^; the RMSE/σ was 1.21, and the R^2^ was −0.94 for *Df* model in randomized test. All models for the randomized test were significantly worse than the models for the original data fits. The results suggest that the models for the original data were physically meaningful.

### 3.3. Dk and Df Exploration

**Figure 6:**
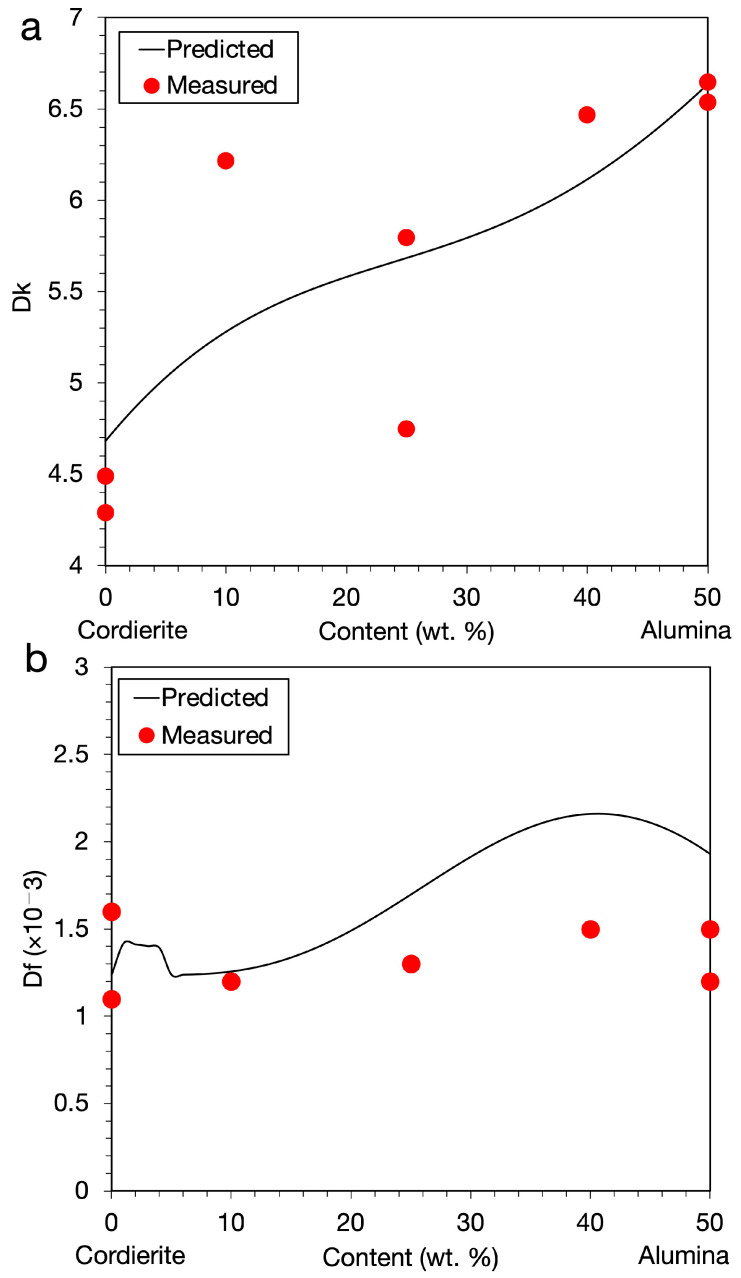
Cross plot of (**a**) *Dk* and (**b**) *Df* against the alumina and cordierite content. MAS was 2 mol%, *T2_R*, *T3_R* (*T2_time*, *T3*_time) were at 1.9979, 1.9982 h/K (1400, 1700 °C × h), and CBSG-S was 50 wt.%. All the other glass phase contents were zero.

Because the data set was rather small and sampling was less homogeneous, it would be difficult to obtain an accurate prediction from extrapolation. Nevertheless, exploring potential LTCC candidates within the composition range of the data set as an interpolation would be still beneficial, especially in the relatively uniform composition spaces shown in Figure 2, e.g., in the alumina, cordierite, and CBSG-S spaces. To see how *Dk* and *Df* evolved along these feature spaces within the data set range, we performed a cross-plot analysis. We chose *T2* = 700 °C, *time_2* = 2 h, *T3* = 850 °C, *time_3* = 2 h (i.e., *T2_R*, and *T3_R* (*T2_time*, and *T3*_time) were set at 1.9979, and 1.9982 h/K (1400, and 1700 °C × h)), CBSG-S = 50 wt.%, MAS = 2 mol%, and all the other glass phases = 0 wt.% for the analysis because a series of LTCCs was fabricated at this range in the data set. Figure 6 shows the cross plot of *Dk* and *Df* against alumina and cordierite content. We found that *Dk* increased when the alumina content increased, as shown in Figure 6a. *Df* in general increased when the alumina content was increased until 40 wt.% and then decreased when alumina content was higher than 40 wt.%, as shown in Figure 6b. A small hump of *Df* increase at the alumina content of 0 to 5 wt.% was also found. Prediction for *Df* was slightly higher than the measured data but the difference was minor. Overall, the trend between the real measurements and the machine learning prediction agreed well with each other. From the cross plots, one would expect to obtain low *Dk* and *Df* values at a low (high) alumina (cordierite) content. 

**Figure 7:**
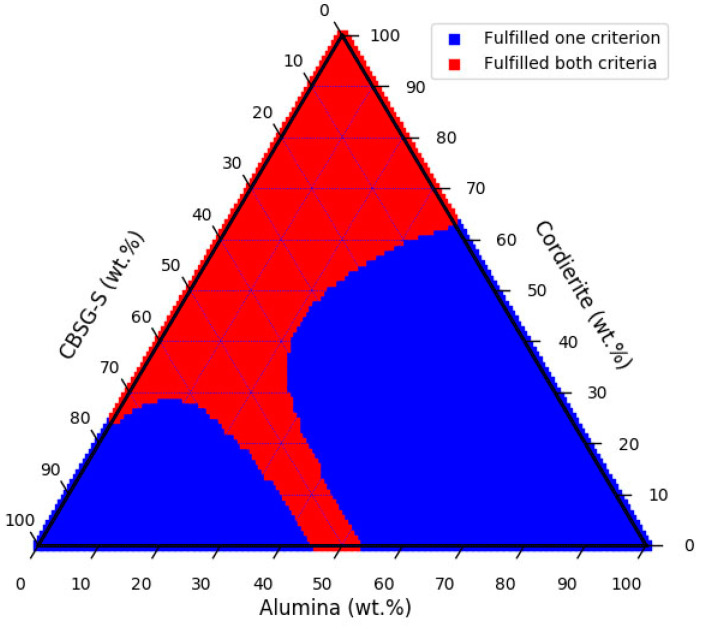
Property contour plot of LTCCs composed of CBSG-S, cordierite, and alumina. The MAS glass was 2 mol%, and the first to third calcination temperatures (time) were 750 °C, 700 °C, and 850 °C (3 h, 2 h, and 2 h), respectively. All the other glass phase contents were zero. The operating frequency was set at 1 GHz.

As the goal for the proposed models is to explore potential LTCCs which have both low *Dk* and *Df* values to control signal delay and energy loss, a property contour plot would be visually useful to define the region fulfilling the given criteria. We followed the criteria provided in Ref [5], i.e., the notion that *Dk* and *Df* should be lower than 10 and 2 × 10^−3^, respectively. We therefore set these criteria for the property contour plot and explored potential candidates within the alumina, cordierite, and CBSG-S content. MAS was again chosen at 2 mol%, and *T2_R*, *T3_R* (*T2_time*, *T3*_time) were set at 1.9979, 1.9982 h/K (1400, 1700 °C × h), respectively. All the other glass phase contents were set at zero. Figure 7 shows the property contour plot. The blue and red regions represent the LTCCs that fulfilled one, and fulfilled both criteria, respectively. This contour plot reveals that if one chooses the compositions in the red region and calcinates them in three different stages, i.e., (1) 750 °C for 3 h, (2) 700 °C for 2h, and then (3) 850 °C for 2 h, these LTCCs will be likely to have *Dk* and *Df* values less than 10 and 2 × 10^−3^, respectively, at an operating frequency of 1 GHz. The plot provides a quick guideline for developing potential LTCCs with low *Dk* and *Df* values, as well as for saving both time and cost. Once more data become available, the proposed models could be further improved and extended to more complex systems.

## 4. Conclusions

In this paper, we built machine learning models for predicting *Dk* and *Df*, and explored potential LTCCs with low *Dk* and *Df* values. Data at an operating frequency of 1 GHz were pulled out from the ITRI-LTCC database to build models. PCA and k-means methods were initially performed to visually analyze data clustering and to reduce the dimension complexity that inherently caused the model to fail. In optimizing the input features, we found that using the calcination reaction product (i.e., *T2_R* and *T3_R*), as well as the calcination temperature and time product (i.e., *T2_time* and *T3*_time), led to a better model performance (i.e., a lower five-fold CV RMSE) as opposed to using temperature and time separately (i.e., *T2*, *T3*, *time_2* and *time_3*) for building *Dk* and *Df* models, respectively. The five-fold CV RMSE was 0.59, the CV RMSE/σ was 0.61, and the R^2^ was 0.57 for the *Dk* model. The CV RMSE was 1.12 × 10^−3^, the CV RMSE/σ was 0.31, and the R^2^ was 0.91 for the *Df* model. CV results suggest that the proposed models captured the complex *Dk* and *Df* properties. Randomized test showed a worse model performance than that for the original data fits. It suggests that the proposed models were not only numerical due to the rather small data set, but were physically meaningful. Cross-plot analysis showed that the machine learning prediction agreed well with the real measurements. Cross-plot analysis suggests that the proposed models had the potential to predict *Dk* and *Df* within the input feature ranges as an interpolation. A property contour plot was built to explore LTCCs for real applications with *Dk* and *Df* values less than 10 and 2 × 10^−3^, respectively, at an operating frequency of 1 GHz. Explorative models were obtained in the current work, and the models can be further improved as new data become available in the future. The proposed machine learning models can potentially be utilized to accelerate the design of LTCCs used in fifth-generation telecommunications.

## Figures and Tables

**Table 1:** The feature information of the initial data set (number of data points = 116).

Feature	Maximum	Minimum	Average	Standard Deviation	Unit	Category
Dielectric constant (*Dk*) at 1 GHz	7.8	2.71	5.33	1	-	Target feature
Dissipation factor (*Df*) at 1 GHz	16.7 × 10^−3^	0.07 × 10^−3^	2.7 × 10^−3^	3.4 × 10^−3^	-	Target feature
Al_2_O_3_ (Alumina)	50	0	8.5	16.1	wt.%	Ceramic filler
Mg_2_Al_4_Si_5_O_18_ (Cordierite)	72.9	0	9.9	19.8	wt.%	Glass-ceramic
Borosilicate glass + filler (BGF)	100	0	3.7	18.4	wt.%	Glass phase
MgO-Al_2_O_3_-SiO_2_ glass (MASG)	100	0	7.7	26.7	wt.%	Glass phase
CaO-B_2_O_3_-SiO_2_ glass (high SiO_2_, CBSG-S)	100	0	25.8	31.6	wt.%	Glass phase
Borosilicate glass (BG)	55	0	6.7	14.4	wt.%	Glass phase
CaO-B_2_O_3_-SiO_2_ glass (high B_2_O_3_, CBSG-B)	100	0	26.5	39.6	wt.%	Glass phase
MgO-Al_2_O_3_-SiO_2_-based ceramic (MAS)	100	0	5.6	15.6	mol%	Glass phase
First stage calcination temperature (*T1*)	1650	27	373.7	386.0	°C	Processing parameter
Second stage calcination temperature (*T2*)	750	27	599.3	263.8	°C	Processing parameter
Third stage calcination temperature (*T3*)	1200	27	852.0	85.6	°C	Processing parameter
First stage calcination time (*time_1*)	3	0	2.7	0.7	h	Processing parameter
Second stage calcination time (*time_2*)	2	0	1.6	0.8	h	Processing parameter
Third stage calcination time (*time_3*)	2	0	1.3	0.8	h	Processing parameter

**Table 2:** The feature information of the data set used in the proposed models (number of data points = 63).

Feature	Maximum	Minimum	Average	Standard Deviation	Unit	Category
Dielectric constant (*Dk*) at 1 GHz	7.8	2.79	5.3	0.97	-	Target feature
Dissipation factor (*Df*) at 1 GHz	17.6 × 10^−3^	0.34 × 10^−3^	2.74 × 10^−3^	3.57 × 10^−3^	-	Target feature
Al_2_O_3_ (Alumina)	50	0	20.16	19.6	wt.%	Ceramic filler
Mg_2_Al_4_Si_5_O_18_ (Cordierite)	70	0	14.29	21.27	wt.%	Glass-ceramic
MgO-Al_2_O_3_-SiO_2_ glass (MASG)	100	0	7.94	27.03	wt.%	Glass phase
CaO-B_2_O_3_-SiO_2_ glass (high SiO_2_, CBSG-S)	100	0	37.6	30.61	wt.%	Glass phase
Borosilicate glass (BG)	55	0	7.72	14.88	wt.%	Glass phase
CaO-B_2_O_3_-SiO_2_ glass (high B_2_O_3_, CBSG-B)	75	0	7.54	19.6	wt.%	Glass phase
MgO-Al_2_O_3_-SiO_2_-based ceramic (MAS)	100	2	8.49	20.54	mol%	Glass phase
Second stage calcination temperature (*T2*)	760	650	711.1	42.5	°C	Processing parameter
Third stage calcination temperature (*T3*)	1200	27	848.8	116.0	°C	Processing parameter
Second stage calcination time (*time_2*)	2	0.5	1.93	0.32	h	Processing parameter
Third stage calcination time (*time_3*)	2	0	1.60	0.71	h	Processing parameter
Second stage calcination reaction product (*T2_R*)	2	0.5	1.93	0.32	h/K	Processing parameter
Third stage calcination reaction product (*T3_R*)	2	0	1.6	0.71	h/K	Processing parameter
Second stage calcination temperature and time product (*T2_time*)	2400	0	1382.54	613.73	°C × h	Processing parameter
Third stage calcination temperature and time product (*T3_time*)	1500	375	1368.65	237.04	°C × h	Processing parameter

**Table 3:** The optimized hyperparameters of *Dk* and *Df* models.

Model	α	γ
*Dk* model	0.0012	0.3530
*Df* model	0.0420	3.2551

## Data Availability

The ITRI-LTCC database is not publicly available and therefore this data is not included in any of the shared files. Requests for the ITRI-LTCC database should be directly sent to Tzu-Yu Liu at jill.t.y.liu@itri.org.tw.
